# Nailfold capillary density in 140 untreated children with juvenile dermatomyositis: an indicator of disease activity

**DOI:** 10.1186/s12969-023-00903-x

**Published:** 2023-10-13

**Authors:** Lauren M. Pachman, Gabrielle Morgan, Marisa S. Klein-Gitelman, Najah Ahsan, Amer Khojah

**Affiliations:** 1 Division of Pediatric Rheumatology, Ann & Robert H. Lurie Children’s Hospital of Chicago, 225 East Chicago Avenue, Box 50, Chicago, IL 60611 USA; 2 Feinberg School of Medicine, Northwestern University, Chicago, IL USA; 3 Department of Pediatrics, College of Medicine, Umm Al-Qura University, Makkah, Saudi Arabia

**Keywords:** Juvenile dermatomyositis, Nailfold vasculature, Myositis specific antibodies

## Abstract

**Background:**

We lack a reliable indicator of disease activity in Juvenile Dermatomyositis (JDM), a rare disease. The goal of this study is to identify the association of nailfold capillary End Row Loop (ERL) loss with disease damage in children with newly diagnosed, untreated JDM.

**Findings:**

We enrolled 140 untreated JDM and 46 age, race and sex matched healthy controls, ages 2–17. We selected items from the Juvenile Myositis Registry for analysis. Variables include average ERL density of 8 fingers, average capillary pattern, hemorrhages, and clinical and laboratory correlates. Laboratory data includes Myositis Specific Antibodies (MSA), disease activity scores (DAS), Childhood Myositis Assessment Scale (CMAS), and standard clinical serologic data. The reduced mean ERL density is 5.1 ± 1.5/mm for untreated JDM vs 7.9 ± 0.9/mm for healthy controls, *p* < 0.0001, and is associated with DAS-skin, *r* = -0.27 *p* = 0.014, which did not change within the age range tested. Untreated JDM with MSA Tif-1-γ had the lowest ERL density, (*p* = 0.037); their ERL patterns were primarily “open” and the presence of hemorrhages in the nailfold matrix was associated with dysphagia (*p* = 0.004).

**Conclusions:**

Decreased JDM ERL density is associated with increased clinical symptoms; nailfold hemorrhages are associated with dysphagia. Duration of untreated disease symptoms and MSA, modify NFC shape. We speculate nailfold characteristics are useful indicators of disease activity in children with JDM before start of therapy.

**Supplementary Information:**

The online version contains supplementary material available at 10.1186/s12969-023-00903-x.

## Background

**Fig. 1:**
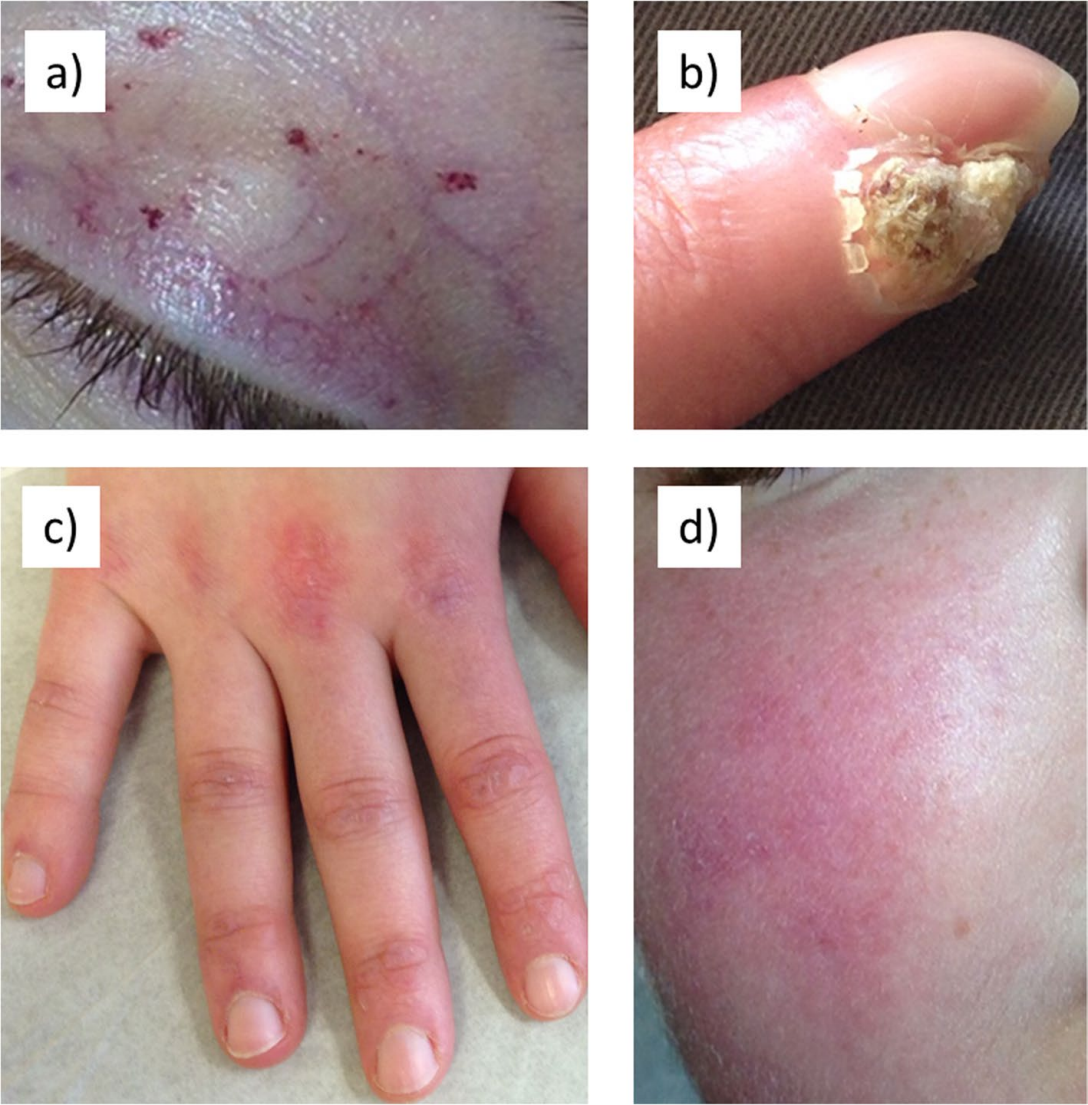
Skin characteristics seen in JDM **a** dilated eyelid capillaries and telangiectasia, **b** calcification of the finger, **c** Gottron’s papules of the hand and **d** erythema of the face

**Fig. 2:**
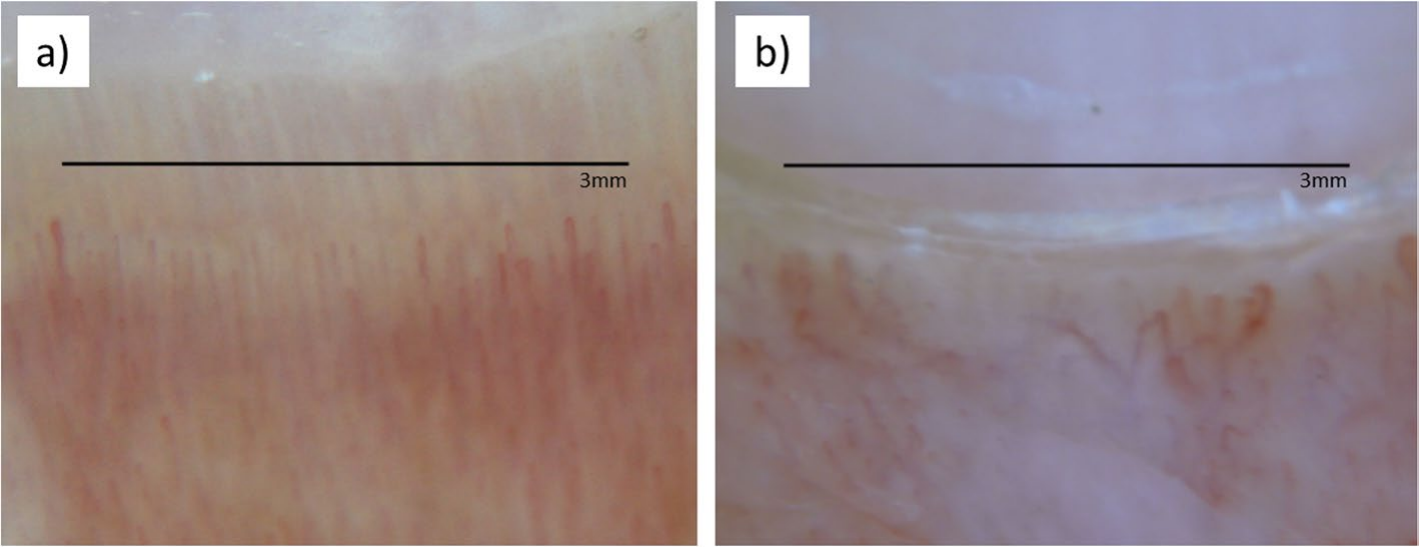
Examples of nailfold capillaroscopy photos of **a** healthy control and **b** untreated Juvenile Dermatomyositis. Scale marker represents 3mm on the nailfold

**Fig. 3:**
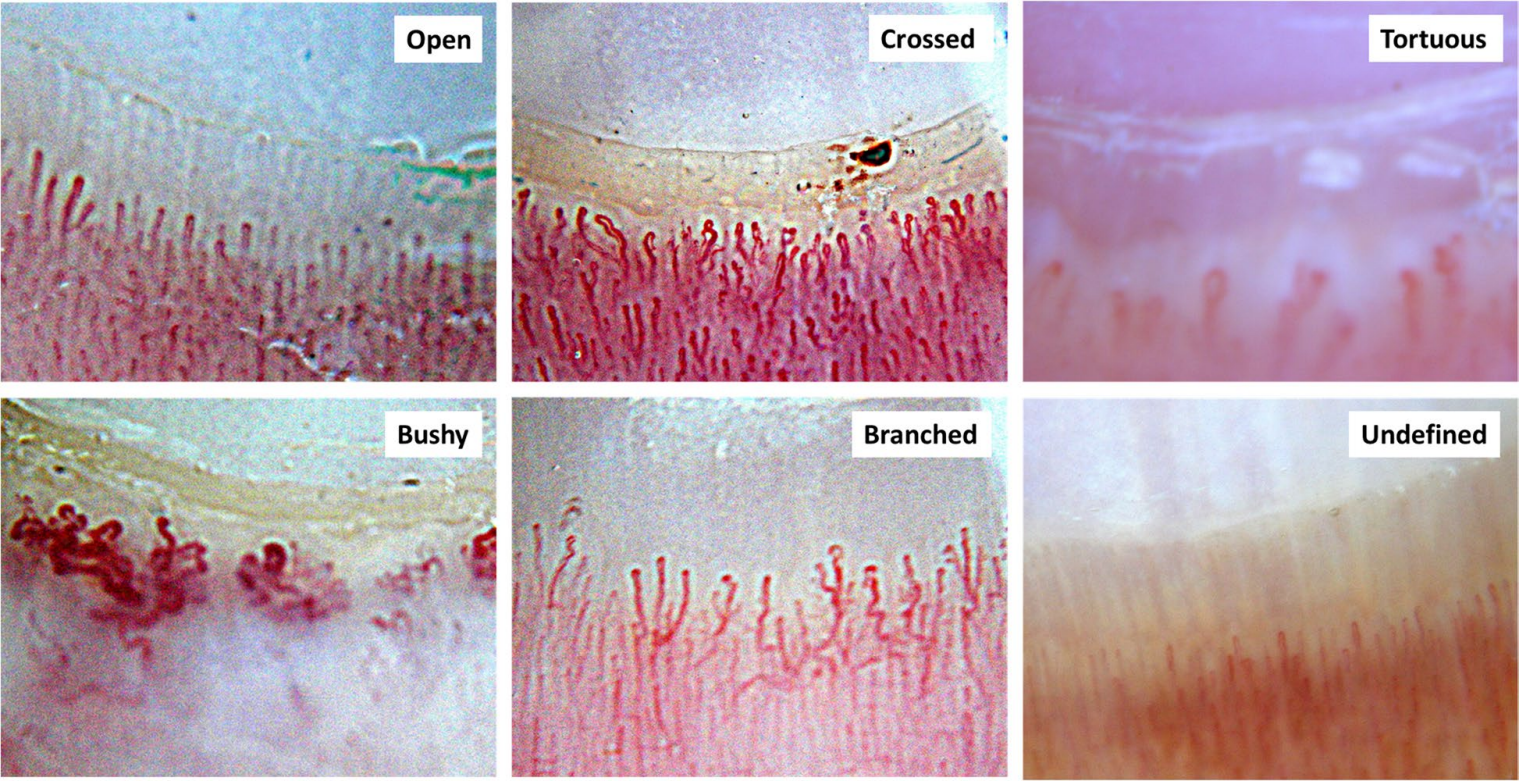
Nailfold capillaroscopy photos of the six most frequent patterns that are easily seen in the ERL of untreated children with Juvenile Dermatomyositis: open, crossed, tortuous, bushy, branched, undefined

Although Juvenile dermatomyositis (JDM) is the most common of the pediatric inflammatory myopathies, it is a rare disease. The annual USA average incidence is 3.2 cases/million children/year white, non-Hispanic, 3.3/million African Americans, and 2.7/million for Hispanic patients, with an overall girl to boy ratio of 2.3 girls:1 boy, and a mean age at JDM diagnosis of 6.7 for girls and 7.3 for boys [1]. Children with JDM have both damage to their vascular system and characteristic inflammatory skin involvement, including periorbital, malar and peripheral erythema, Gottron’s papules, and in some cases, calcification (Fig. 1). In addition, they often display dysphagia and proximal muscle damage, accompanied by focal weakness. For example, weakness may be initially manifest as difficulty in climbing stairs or getting in/out of the car, or trouble with swallowing. In addition to the musculoskeletal damage, examination of the capillary loops in the nailbeds of the child with active JDM shows that these capillary loops are both decreased in number, compared with healthy age-matched controls, and that they are misshapen (Fig. 2). The capillary loops may be dilated, shrunken or deleted and free blood—hemorrhages–may be present in the nailfold area as well (Fig. 3).

Over 30 years ago, we created the Ann & Robert H. Lurie Children’s Hospital of Chicago Juvenile Myositis Registry and Repository. Our recent RNA sequencing (RNA-Seq) of peripheral blood mononuclear cells, as well as skin and muscle biopsies samples from, untreated JDM, each demonstrated the presence of robust transcriptional activity both at diagnosis and when they appeared to be “clinically inactive” [2]. These RNA-Seq data supported our previous serologic evidence of inflammatory disease activity in the apparently clinically quiescent JDM [3]. This observation led to the speculation that data pertaining to the decreased density of nailfold capillary (NFC) endrow loop (ERL) may provide a qualtitative estimate of disease activity sufficient to guide to therapy for children with JDM [4, 5].

## Findings

### Methods

#### Patient population

Untreated JDM (*n* = 140), who met diagnosis of JDM [6], and 1:3 ratio of age, sex, race matched healthy controls (*n* = 46) gave written, age-appropriate informed consent (Ann & Robert H. Lurie Children’s Hospital of Chicago IRB# 2010–14117, 2001–11715, 2011–14651) (Table 1). Written informed consent was obtained from all legally authorized representatives and assent from those patients aged 12 and older. The children with JDM were diagnosed and seen at Lurie Children’s between 1993 and 2021, and had proximal muscle weakness, the characteristic rash, varying degrees of elevated muscle enzymes and, since 2000, a T2 weighted MRI image of involved muscle. Children with overlap syndrome (positive anti-U1 RNP, anti-U2 RNP or anti-PM-Scl), Systemic Lupus or Scleroderma were excluded.

**Table 1:** Demographics of untreated JDM and healthy controls

	JDM	Healthy Control	*P*-value
	*n* = 140	*n* = 46	
Demographics			
Age, mean (SD)	6.7 (3.7)	7.7 (3.5)	0.125
Sex, n (%)			
Female	111 (79.3)	35 (76.0)	0.647
Male	29 (20.7)	11 (24.0)	
Race, n (%)			
White, Non-Hispanic	105 (75.0)	31 (67.4)	0.102
White, Hispanic	24 (17.1)	7 (15.2)	
Black	5 (3.6)	2 (4.4)	
Asian	4 (2.9)	1 (2.2)	
Other	2 (1.4)	4 (8.7)	
Unknown	0 (0)	1 (2.2)	
Capillary End Row Loop (ERL)density (x/mm), mean (SD)	5.1 (1.5)	7.9 (0.9)	** < 0.0001**

#### Clinical assessments

Routine clinical laboratory tests were performed in the Lurie Children’s diagnostic laboratories, while The Oklahoma Medical Research Foundation assayed the Myositis Specific Antibodies (MSA), using immune precipitation and immunodiffusion [7]. Their early MSA data were first available after 2002, and the P155/140 (Tif-1-γ), MJ (NXP-2), and MDA5 (anti-CADM140) antibodies were reported after 2012. Of the 140 untreated JDM in this study, 94 had current MSAs, including these newly reported antibodies. The Disease Activity Score (DAS) was obtained by a pediatric rheumatologist; DAS-total score (DAS-T) (range = 0–20), is derived by adding the muscle evaluation (DAS-M) (range = 0–11) to the skin score (DAS-S) (range = 0–9) [8]. A physical therapist obtained The Childhood Muscle Assessment Scale (CMAS) [9]. The duration of untreated disease (DUD) is defined as the length of time that the parents/caregiver first noted either new physical signs of JDM or change in activity to the date of the first JDM treatment.

#### Nailfold capillaroscopy

Since 2012, a digital camera (Nikon Coolpix p6000) equipped with a Dermlite2 ProHR provided standardized NFC images (18x) to generate the data and assess inter-rater reliability, prior to 2012, freeze frame videomicroscopy was utilitized as previously described [5]. Figure 2 illustrates the difference seen in healthy control and untreated JDM periungual NFCs. Qualitative measures, such as severity of avascularity, and predominant ERL shape are entered on the NFC work sheet (Table 2). The main patterns of ERLs are open, undefined, crossed, bushy, branched, and tortuous; the predominant pattern was recorded, Fig. 3. The reproducibility of the method was assessed by two experienced readers who analyzed the images of 49 JDM utilizing Photoshop –see accompanying 3-min video of the method (attachment).

**Table 2:** Assessment score sheet for ERL in children with JDM and their controls

ID #: _____________		Name: ____________________					Date of Exam: ____/____/____			
		**Right Hand**					**Left Hand**			
		**2**^**nd**^	**3**^**rd**^		**4**^**th**^	**5**^**th**^	**2**^**nd**^	**3**^**rd**^	**4**^**th**^	**5**^**th**^
**Total number of loops /3mm**										
**Calculated number loops /mm** (x total number of loops/3)										
**Number dilated /3 mm:**										
**Severity of dilation:** 1 = mild, 2 = moderate, 3 = severe										
**Avascular Pattern:** 1 = periungual, 2 = patchy, 3 = both										
**Severity of avascularity:** 1 = mild, 2 = moderate, 3 = severe										
**Predominant pattern:**										
1 = open	2 = crossed									
3 = tortuous	4 = undefined									
5 = bushy	6 = bushy									
**Number bushy loops /3mm:**										
**Number Branched Points /3mm:**										
**Number of Hemorrhages (any):**										
**GLOBAL**										
** Avg. end row loops /mm:**										
** Impression:** 1 = Normal 2 = Abnormal										
Date entered ___/___/___ by (initials)____				Date verified ___/___/___ by (initials)____						

#### Statistical analysis

The association of a panel of JDM clinical factors with the NFC data was assessed using Pearson’s correlation co-efficient, correcting for number of comparisons made utilizing the Bonferonni correction. Standard t-tests were employed on other occasions. The association of the shape of the nailfold capillary ERL with the child’s MSA was determined by Chi-square analysis. The statistics were performed in SPSS and figures were generated using Graphpad Prism 9 software.

### Results

#### Inter-rater reliability for NFC analysis

Two trained observers, assessing nailfold data from 49 children with JDM were highly correlated for ERL/mm counts (*r* = 0.817, *p* =  < 0.0001).

#### NFC studies of 140 untreated JDM and 46 matched healthy controls

**Fig. 4:**
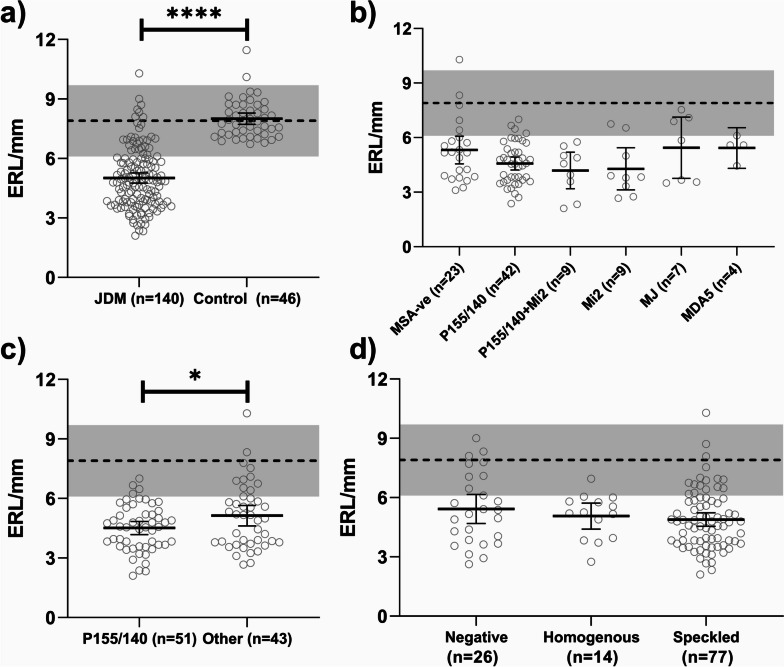
**a** Nailfold capillary end row loop (ERL) density in 140 untreated children with JDM and 46 healthy controls. The dotted line is the mean ± 2 standard deviations (shaded area) for controls. The mean ERL for JDM was 5.0 ± 1.5/mm, vs the mean ERL for healthy controls, 8.0 ± 0.9/mm, *p* < 0.0001. **b** The association of a range of MSA with nailfold end row loop capillary density in 94 untreated children with JDM. **c** Comparison of nailfold end row loop capillary density for 51 untreated children with JDM positive for P155/140 MSA with 43 untreated JDM positive for other MSA or MSA negative, * = significance at 0.05. **d** The lack of association of a positive antinuclear antibody with a significant difference in the density of the nailfold capillary end row loops

In general, the JDM patients had moderate disease activity at the time of their first nailfold photography; the mean DAS-T = 10.8 ± 3.3 SD; (DAS-S = 5.7 ± 1.3; DAS-M = 5.1 ± 2.8); the von Willebrand Factor Antigen (vWF:Ag) (corrected for blood group antigen) was elevated in 24% (mean 159.6 ± 88.0%) of untreated JDM (Table 3). JDM patients had fewer ERLs than healthy controls (5.1 ± 1.5/mm vs 7.9 ± 0.9/mm, *p* < 0.0001, Table 1, Fig. 4a). Neither group had a significant association of ERL density with sex (JDM: *p* = 0.277, healthy control: *p* = 0.98) or age (JDM: *r* = 0.096, *p* = 0.99, healthy control: *r* = -0.211, *p* = 0.16). The longer the duration of untreated disease, the more damage to the ERL: (*r* = -0.174, *p* = 0.28). For the entire untreated JDM group, the children with decreased ERL had more skin symptoms than muscle findings (DAS-S: *r* = -0.267, *p* = 0.014 vs DAS-M: *r* = 0.032, *p* = 0.99).

**Table 3:** Correlations of clinical factors and end row capillary loops in untreated JDM

	mean (SD)	Pearson Correlation Coefficient	*P*-value^b^
Age, years	6.7 (3.7)	0.096	0.99
Duration of Untreated Disease (DUD), months	9.1 (12.4)	-0.174	0.32
Childhood Myosotis Assessment Scale (CMAS)^a^ (*n* = 86)	32.0 (13.2)	0.045	0.99
Disease Activity Score (DAS)-Total, (*n* = 136)	10.8 (3.3)	- 0.077	0.99
DAS-Skin, (*n* = 137)	**5.7 (1.3)**	**- 0.267**	**0.014**
DAS-Muscle, (*n* = 139)	5.1 (2.8)	0.032	0.99
von Willebrand Factor Antigen % (vWF:Ag), (*n* = 124)	**159.6 (88.0)**	**0.242**	**0.049**

^a^CMAS was initiated in our clinic setting in 2002

^b^Bonferroni Correction

With respect to the MSAs, 44.7% of the children had anti-P155/140 (anti-TIF1-γ), 9.6% anti-Mi2, 9.6% multiple MSA [anti-P155/140 (anti-TIF1-γ), anti-Mi2], 7.4% anti-MJ (anti-NXP-2), 4.3% anti-MDA5 (anti-CADM140), and 24.5% MSA negative JDM, Fig. 4b**.** P155/140 (anti-TIF-1-γ) was associated with lower ERL density. MJ (NXP-2) antibody was associated with a wider, but still abnormal data range (Fig. 3b). When the anti-P155/140 (anti-Tif-1-γ) group (including children who tested positive for both anti-P155/140 and anti-Mi2) were compared with the aggregated data for the other JDM MSA types, the difference was significant (*p* = 0. 037), Fig. 4c. The ERL were not associated with a positive anti-nuclear antibody, Fig. 4d.

**Fig. 5:**
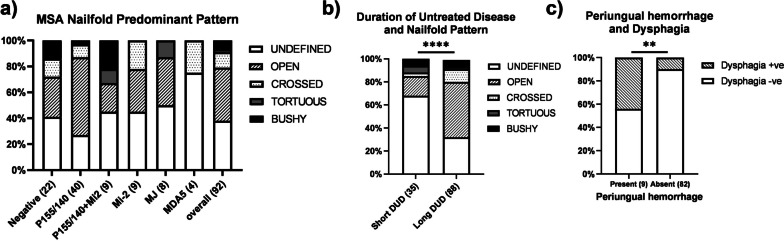
**a** Distribution of predominant ERL pattern in untreated JDM patients by various MSAs **b** Untreated children with short DUD have more undefined patterns than long DUD group (Chi-square, *p* =  < 0.0001). **c** Untreated children presenting with dysphagia have more periungual hemorrhages present in their nailfold capillaries than those not presenting with dysphagia (Chi-square, *p* = 0.004)

This study was initiated approximately 25 years before information about the specificity of the MSAs were identified [10]; 33% of the early cases of JDM in this study did not have current MSA data. Assessment of 92 JDM with both current MSA and ERL pattern data disclosed that their initial patterns were: 41% open, 38% undefined, 12% crossed, 7% bushy, and 2% tortuous (Fig. 5a). When the JDM were grouped by their MSAs, those with P155/140 had a predominant ERL pattern that was more “open” than the patterns in the other groups– MSA negative, MSA Mi-2 combined with P155/140, and all other MSA (*p* = 0.03). As Fig. 5b presents, the u*ndefined* pattern was associated with the shorter DUD, with a median of 3.6 months in comparison to those with a *crossed* shape (median of 10.6 months) or an o*pen* shape (median 5.8 months), (Kuskal-Wallis test, *p* = 0.036). The *undefined* pattern was also associated with the highest ERL capillary density with a median of 5.6/mm in comparison to 3.8/mm for the c*rossed* group and 4.2/mm for the *open* group (Kruskal–Wallis test, *p* = 0.002). Furthermore, when DUD was dichotomized into either a short (≤ 3 months) and long (> 3 months) duration, those children having a shorter DUD had more *undefined* predominant NFC pattern than those with a longer untreated disease duration (*p* =  < 0.0001), suggesting progression over time, Fig. 5b. Of note, 13% of the study group had *periungual* hemorrhages on their first nailfold assessment, which were closely associated with the symptoms of dysphagia (Chi-Square, *p* = 0.004) (Fig. 5c).

## Discussion

Twenty-five percent of our untreated JDM are small children with a short attention span, aged 4 or younger at diagnosis [11], making speed and accuracy essential components of this nailfold capillaroscopy method. The method of obtaining the images described in the accompanying video is both useful and reproducible. When the interrater reliability of trained personnel was obtained for 49 JDM, the two readers’ data were correlated (*r* = 0.817, *p* =  < 0.0001) similar to previous reports of nailfold capillary data for adults with Scleroderma [12]. Our data concentrates on ERL number and shape, not rate of capillary blood flow. These data also document that, children with the MSA P155/140 (Tif-1-γ) have a significantly greater loss of ERL when compared with all the other MSAs combined, Fig. 4c. Figure 5a illustrates that the open loop shape is the most prevalent in JDM positive for MSA, P155/140 (Tif-1-γ), while Fig. 5b documents that an undefined pattern is associated with a shorter JDM disease duration. The capillaries are specific targets of the inflammatory process in JDM muscle [13]; capillary injury preceeds muscle fiber damage. With respect to the intestine, ERL density is highly associated with the bioavailability of some drugs used in inflammatory bowel disease—Crohn’s disease or ulcerative colitis. JDM with reduction in their nailfold capillary ERL may also have weight loss [14]. The vWF:Ag is elevated in 24% of untreated JDM *p* = 0.006 [15]; serum ICAM-1 is increased as well [16].

NFC morphology in patients with JDM is often indistinguishable from children with Scleroderma, but the presence of “bushy loops” is specific for dermatomyositis. Ongoing investigations on the application of artificial intelligence (AI) for analyses of nailfold capillaroscopy images have promising prospects. Standard AI models (such as the Efficient Net deep neural network architecture) can discriminate between NFCs from patients with JDM and healthy controls [17]. We have presented the critical and innovative observation that the nailfold ERL density may reflect gastrointestinal function [18]–which can influence both the optimal type and route of immunosuppressive therapy. The weaknesses of this study include the inherent inter-rater variation.

## Conclusion

This report provides comprehensive data at diagnosis for a large group of untreated children with a rare disease, JDM, seen by consistent personnel at a single center. The score sheet provides a simple data collection tool, while the short methods video provides photographic guidance to the collection of these data. We can now conclude that decreased nailfold capillary density is more associated with cutaneous disease activity than muscle involvement at JDM diagnosis. This study documents that nailfold hemorrhages are associated with dysphagia, an often overlooked, but critical dysfunction. Finally, we now have evidence that the ERL shape is modified both by the child’s MSA, as well as the duration of their untreated symptoms.

### Supplementary Information

**Additional file 1.**

## Data Availability

The dataset analyzed for this study are available from the corresponding author on reasonable request.

## Abbreviations

JDM: Juvenile Dermatomyositis
RNA-Seq: RNA sequencing
NFC: Nailfold capillary
ERL: End row loops
MSA: Myositis-specific antibodies
DAS: Disease Activity Score
DAS-T: Disease Activity Score total
DAS-M: Disease Activity Score muscle compoments
DAS-S: Disease Activity Score skin components
CMAS: Childhood Myositis Assessment Scale
DUD: Duration of untreated disease
vWF:Ag: Von Willebrand factor antigen
AI: Artificial intelligence
